# Supplementary material for: ISG15 Is a Novel Regulator of Lipid Metabolism during Vaccinia Virus Infection
Source: Microbiol Spectr. 2022 Dec 1;10(6):e03893-22. doi: 10.1128/spectrum.03893-22 (PMC9769738; doi:10.1128/spectrum.03893-22)
Supplement: Supplemental file 1 — Figure S1. Download spectrum.03893-22-s0001.pdf, PDF file, 0.5 MB [file spectrum.03893-22-s0001.pdf]

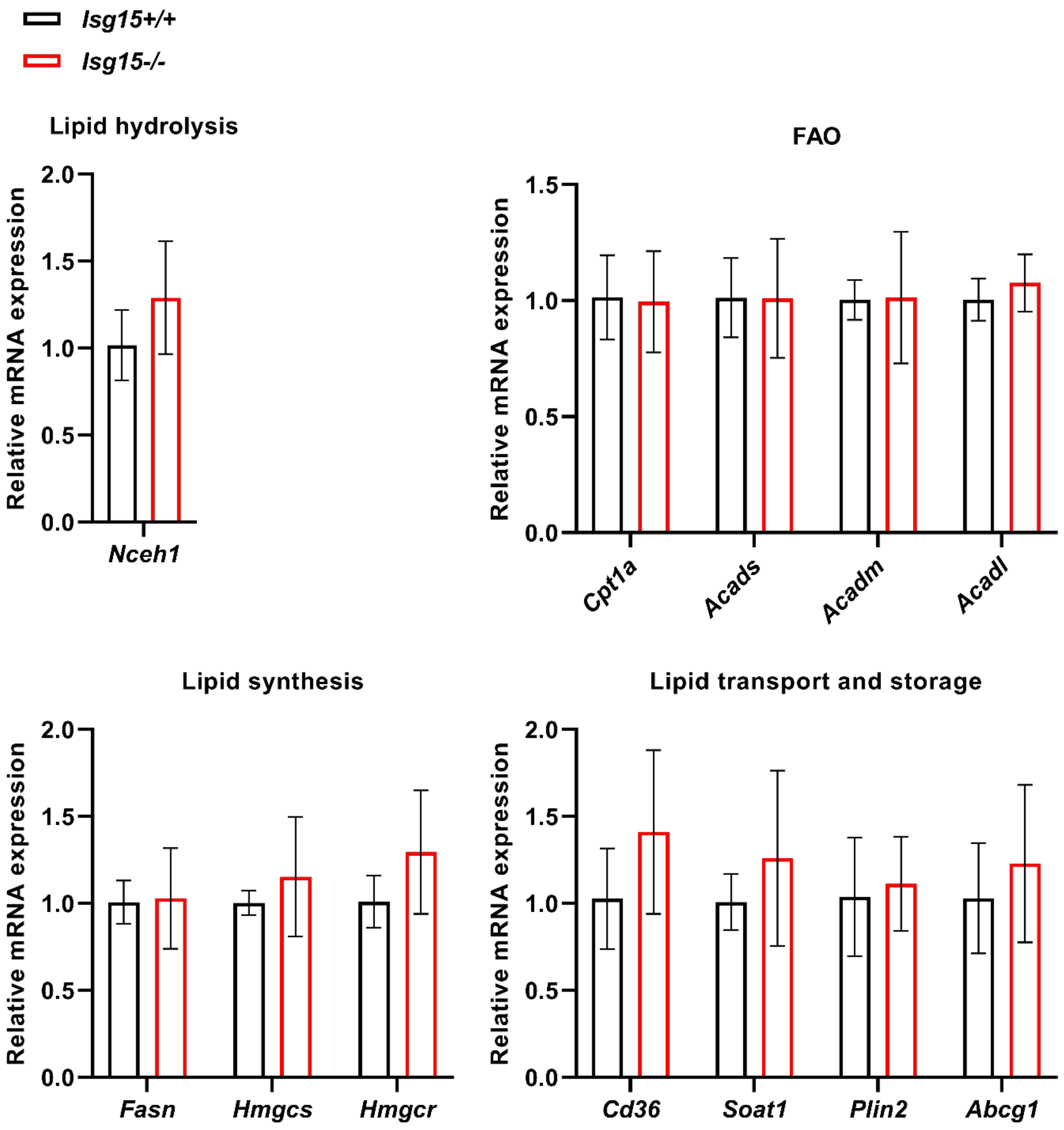

Figure S1. Analysis of the expression of lipid metabolism genes and metabolic regulators in Isg15-/- and WT BMDM. The expression levels of key genes FAO, lipid synthesis and lipid hydrolysis are similar between *Isg15-/-* and WT BMDM. mRNA levels of the indicated genes were analyzed by RT-qPCR in IFN-treated *Isg15-/-* and WT BMDM. Expression levels were normalized to HPRT mRNA levels. Mean ± SD of 3 biological replicates is represented. Student's t-test or Welch's t-test were used for the comparisons. * *p-value* < 0.05; ** *p-value* < 0.01: *** *p-value* <0.001.

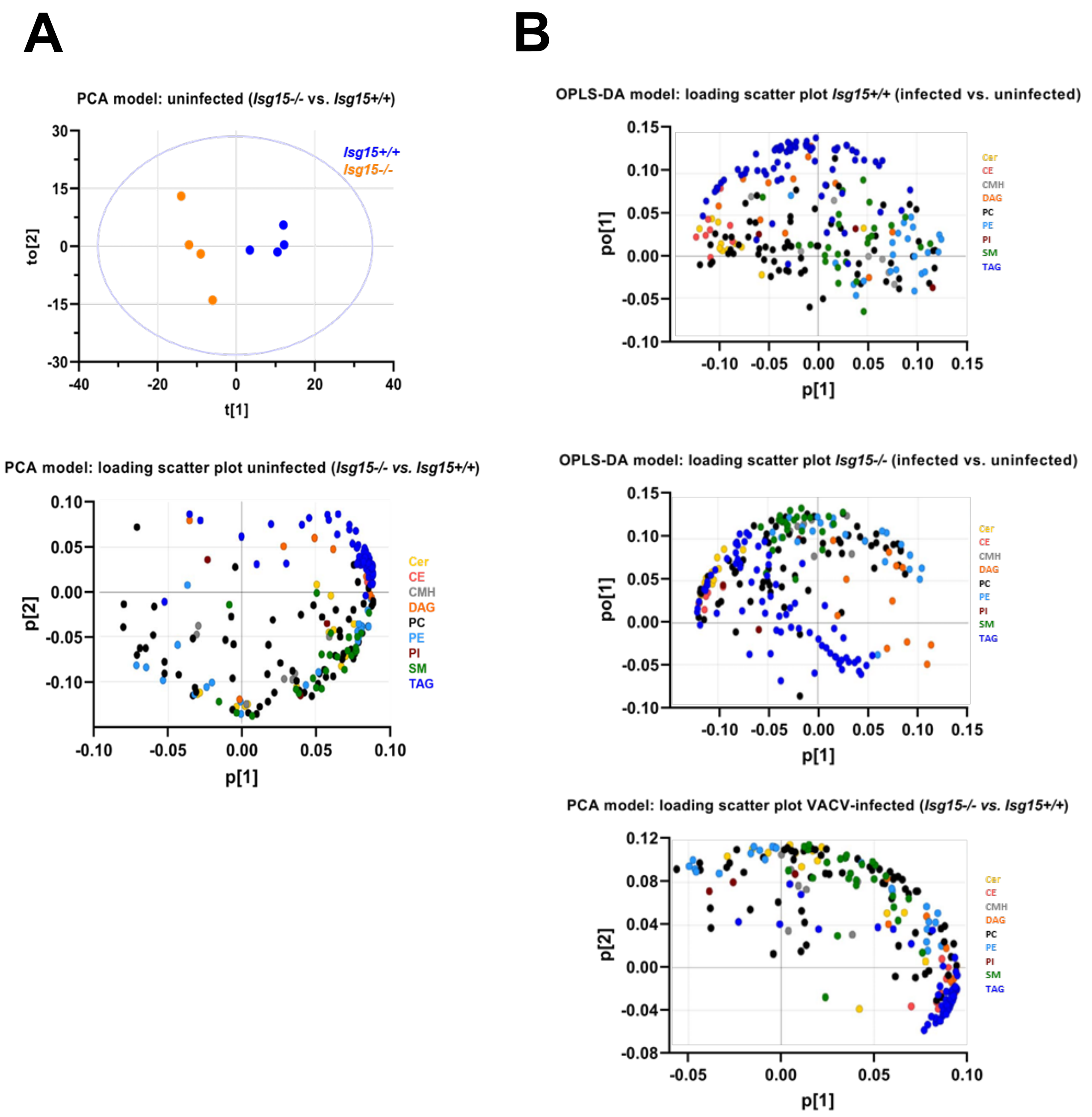

Figure S2. **Lipidomic analysis of uninfected and VACV-infected *Isg15-/-* and WT BMDM. Sample quality control. (A).** Principal component analysis (PCA) was performed with samples of uninfected BMDM to evaluate the clustering between samples. Clustering at the sample (upper panel) and metabolite (low panel) levels is represented. **(B).** Principal component analysis and orthogonal partial least-squares to latent structures (OPLS) analysis were performed with samples of VACV-infected BMDM to evaluate the clustering between samples. Clustering at the metabolite level is represented.

Table S1. **Antibodies used for western blot analysis.**

Antibody	Source
β-ACTIN	Cell Signaling #3700
ISG15	Invitrogen (14-5758-82)
NCEH1	Sigma-Aldrich SAB4301148
Anti-rabbit IgG-peroxidase	Sigma-Aldrich A0545
Anti-mouse IgG-peroxidase	Sigma-Aldrich A9044
Anti-Armenian hamster IgG-peroxidase	Sigma-Aldrich SAB3700454

Table S2. Murine primers used in RT-qPCR analysis of mRNA expression.

Gene	Forward primer (5'-3')	Reverse primer (5'-3')
Abcg1	CCTTCCTCAGCATCATGCG	CCGATCCCAATGTGCGA
Acadl	TCTTGCGATCAGCTCTTTCA	GGTACATGTGGGAGTACCCG
Acadm	AGCTCTAGACGAAGCCACGA	GCGAGCAGAAATGAAACTCC
Acads	AGCTGGACAGGGAGCATCT	ACTCAGCTCCTCTGGCACAT
Cd36	GCGACATGATTAATGGCACA	CCTGCAAATGTCAGAGGAAA
Cpt1a	GATGAACTTCTTCTTCCAGGAGTGC	ATGGCAGAGGCTCACCAAGC
Fasn	AAGTTGCCCGAGTCAGAGAACC	ATCCATAGAGCCCAGCCTTCCATC
Hmgcr	CTTGTGGAATGCCTTGTGATTG	AGCCGAAGCAGCACATGAT
Hmgcs	GCCGTGAACTGGGTCGAA	GCATATATAGCAATGTCTCCTGCAA
Hprt	GATTAGCGATGATGAACCAGGTT	CCTCCCATCTCCTTCTTCATGACA
Isg15	TGACTGTGAGAGCAAGCAGC	CCCCAGCATCTTCACCTTTA
Nceh1	CGGTATTTCTGGAGACAGTGCTG	GGTGTGTTGAAGTCCAAAGCCTG
Nr1h3	TGAGAGCATCACCTTCCTCA	TGGAGAACTAAAGATGGGG
Plin2	CCCGTATTTGAGATCCGTGT	CAATTTGTGGCTCCAGCTTC
Ppara	GCGGCCCCATACAGGAGAGCAG	CTAACCTTGGGCCACACCTTGACT
Pparg	TCGCTGATGCACTGCCTATG	GAGAGGTCCACAGAGCTGATT
Ppargc1a	TATGGAGTGACATAGAGTGTGCT	CCACTTCAATCCACCCAGAAAG
Ppargc1b	TGCGGAGACACAGATGAAGA	GGCTTGTATGGAGGTGTGGT
Scap	ATTTGCTCACCGTGGAGATGTT	GAAGTCATCCAGGCCACTACTAATG
Soat1	AGCCCAGAAAAATTTCATGGACACATACAG	CCCTTGTTCTGGAGGTGCTCTCAGATCTTT
Srebf2	GCGTTCTGGAGACCATGGA	ACAAAGTTGCTCTGAAAACAAATCA
Tfam	AATTGCAGCCATGTGGAGGGA	GCTCTCAGGTGGGATGCAG
VACV	CATCATCTGGAATTGTCACTACTAAA	ACGGCCGACAATATAATTAATGC
